# Characteristics of intussusception among children in Korea: a nationwide epidemiological study

**DOI:** 10.1186/s12887-019-1592-6

**Published:** 2019-06-28

**Authors:** Soojin Jo, In Seok Lim, Soo Ahn Chae, Sin Weon Yun, Na Mi Lee, Su Yeong Kim, Dae Yong Yi

**Affiliations:** 1 Department of Pediatrics, Chung-Ang University Hospital, 102, Heukseok-ro, Dongjak-gu, Seoul, 06973 Republic of Korea; 2 College of Medicine, Chung-Ang University, Seoul, Republic of Korea

**Keywords:** Intussusception, Child, Epidemiology, Lymphadenitis

## Abstract

**Background:**

Intussusception is a gastrointestinal condition in which early treatment is critical. Although its epidemiology and comorbidities have been studied, few studies have included the entire pediatric population of a country. Therefore, we aimed to analyze the epidemiologic features of pediatric intussusception patients and identify comorbidities associated with intussusception in South Korea, using the public health database.

**Methods:**

We analyzed the data of children below 18 years of age, from the national database of South Korea, who were diagnosed with intussusception and managed such as air reduction or surgical methods from 2008 to 2016. Patients were categorized into six groups based on the comorbid diseases. Patients with structural lesion in gastrointestinal tract were divided diagnosis or diagnosis code.

**Results:**

The number of patients diagnosed with intussusception were 25,023 (16,024 males, 64.0%). Of them, the highest percentage was patients aged between 2 and 36 months (20,703; 82.7%). The incidence per 100,000 individuals aged up to 2 years was 196.7. The number of males were 16,024 (64.0%) and were almost twice the number of 8999 (36.0%) female patients. The maximum number of cases (*n* = 2517; 10.1%) were seen in September, followed by July (*n* = 2469; 9.9%). In February, the number of cases was lowest at 1448 (5.8%) patients (*P* < 0.001). The number of patients with structural lesions of the gastrointestinal tract that could lead to intussusception was 1207 (4.8%), while patients with acute gastrointestinal infectious disease were 4541 (18.1%). Among the structural lesions of the gastrointestinal tract that could be the leading cause of intussusception, lymphadenopathy was the most common, seen in 462 (56.6%) patients and an appendix-related condition was seen in 260 (31.9%) patients. Infectious diseases were more common in the younger children, while systemic diseases were more common in the older.

**Conclusion:**

We confirmed that pediatric intussusception in South Korea shows a seasonal tendency, which is age-dependent and is associated with an exposure to infectious agents. Some infectious pathogens and underlying diseases might play an important role in the pathophysiology of intussusception.

## Background

Intussusception is the invagination of a segment of the bowel into the distal part of the bowel, and is the most common cause of obstruction of the intestine in infants [1, 2]. It is known to occur in 74 per 100,000 people, but varies from 9 to 328 per 100,000 people depending on the geography and as reported in different researches. No seasonal pattern for the disease is known yet [3].

Due to the characteristic clinical findings and awareness about the condition, a relatively early screening performed using ultrasonography [4]. However, if the diagnosis is delayed beyond 48 h, complications and mortality could be higher [5, 6]. Hence, early diagnosis and treatment is important. In most cases, intussusception is known to be idiopathic without any underlying disease [7]. However, idiopathic intussusception can also occur after respiratory or gastrointestinal infections, and is associated with various diseases such as Henoch-Schönlein purpura (HSP), lymphoma, Meckel’s diverticulitis, and polyps [7–9]. Although it is not known to be related to natural rotavirus infections, there have been previous reports of intussusception following rotavirus vaccines. Adenovirus infection is known to be associated with intussusception [10, 11]. Many studies have been conducted on the association of intussusception with these specific infections and are well known in relation to structural leading points [12, 13]. However, there have been few studies based on the national health database about the association of other diseases with intussusception [3]. Moreover, there are few studies based on the national health database about the epidemiology of intussusception itself, and there has been no study conducted in Korea at all.

Hence, we aimed to analyze the epidemiologic features of pediatric intussusception using the public health database. We also identified comorbidities associated with intussusception.

## Methods

### Data extraction of pediatric intussusception patients

South Korea has an universal Health Insurance Review and Assessment Service (HIRA) system, which covers more than 98% of the population [14]. The HIRA database contains information such as hospitalization, diagnosis, and medication for almost the entire population of South Korea. Open data is available for research purposes through a rigorous screening process, without any personal information about the patients being disclosed. A personal identification number is provided with restricted personal information, which can be used to extract clinical data such as diagnosis, medication, surgery, and treatment, and demographic information such as age, sex, and geography for each patient. We extracted the data of patients below 18 years of age diagnosed with intussusception (K561) from January 2007 to December 2016, according to the International Classification of Disease (ICD) 10 codes from this open HIRA database.

However, there is a possibility of over diagnosis in the HIRA database, as also a probability of missed cases of actual intussusception. Since the HIRA database captures the input from each medical institution, a case might be included twice if it is diagnosed at one medical institution and transferred to another institution. Additionally, when intussusception is suspected but not confirmed, it might still be included in the database if indicated as a diagnosis by the medical institution while entering the data. Moreover, in cases of suspected small bowel intussusception, some patients do not require any treatment, but are only kept under observation. However, if intussusception is confirmed, air reduction or surgical methods such as manual reduction or right hemicolectomy needs to be performed. Hence, we selected only patients who underwent procedures such as air reduction or surgical methods for treatment of intussusception. Records from 2007 were screened to identify cases of previous diagnosis with intussusception. Data of all patients below 18 years of age in Korea who were treated for intussusception from 2008 to 2016 were examined.

Infants and children below 6 years of age were categorized into early childhood, while those above 6 years of age were classified into middle childhood (6–12 years of age) and early adolescence (12–18 years) according to the stages described by the Eunice Kennedy Shriver National Institute of Child Health and Human Development [15].

Data analysis and statistical analysis were performed using SAS (version 9.2) in the HIRA system. Statistical analysis was performed using the SPSS 18.0 statistical software (SPSS Inc., Chicago, IL, USA).

### Classification of diseases associated with intussusception

Patients diagnosed and treated for intussusception were retrospectively categorized into six groups based on the comorbid diseases, using the diagnosis mentioned in HIRA and ICD-10 codes; Group I: Structural lesion in the gastrointestinal tract, Group II: Acute gastrointestinal infectious disease (A02, A04, A08, A09, and so on), Group III: Acute respiratory infectious disease (J00-J42), Group IV: Other infectious disease (A41, B08, B17, B18, B34, H10, H65, H66, K12, N30, and so on), Group V: Systemic non-infectious disease (D50, D6, D7, E16, and so on) group VI: No accompanying disease.

Group I was divided based on lymphadenopathy into mesenteric lymphadenitis (I880, I881, I889, and I899), appendix related condition (K352, K353, K358, K36, K37, K380, K381, K388, and K389) including appendicitis and appendicolith, tumor (C778, C837, C859, D120, D122, D123, D126, D131, D133, D139, and D201) such as lymphoma and benign neoplasm, HSP (D690), diverticulitis (K571, K573, K574, and K579), and polyp (K635) by diagnosis or diagnosis code.

## Results

### Epidemiology of pediatric intussusception

In the data extracted from the HIRA database between 2007 and 2016, a total of 49,315 patients under the age of 18 were diagnosed with intussusception. Of these, 25,023 patients who were diagnosed with intussusception during the 9 years from 2008 to 2016, and underwent air reduction or surgical treatment such as right hemicolectomy were included. Of these patients, 16,205 (64.8%) were hospitalized and 6 (0.02%) patients died. Surgery was performed in 796 patients (3.18%) due to the failure of reduction. The number of males was 16,024 (64.0%), which was almost twice the number of 8999 (36.0%) female patients (Table 1). The highest number of cases 3389 (13.5%) occurred in 2010 and the lowest number of cases were 2420 (9.7%) in 2015 (*P* < 0.001). Seasonally, the largest number of cases (*n* = 2517; 10.1%) was seen in September, followed by July (*n* = 2469, 9.9%), and August (*n* = 2445, 9.8%). In February, the number of cases was lowest at 1448 (5.8%) patients (*P* < 0.001). Compared to the other seasons, a relatively higher number of patients was treated in August and September 2010. Hence, the number of patients 2171 (10.0%) were the highest in July, during the period of 8 years excluding 2010. The occurrence was more common during the warm season from June to August, with 7218 cases (28.8%) while 5041 (20.1%) cases in the cold season were recorded.

**Table 1:** Number of patients according to year, month, and gender in children treated for intussusception in Korea

	2008	2009	2010	2011	2012	2013	2014	2015	2016	Total number of patients
Month										
January	245	203	148	191	190	218	183	156	225	1759 (7.0%)
February	191	164	144	151	170	149	135	167	177	1448 (5.8%)
March	201	235	211	219	189	157	177	169	189	1747 (7.0%)
April	219	234	235	256	231	205	220	198	175	1973 (7.9%)
May	252	256	272	264	258	298	226	240	280	2346 (9.4%)
June	280	255	272	265	250	283	218	180	301	2304 (9.2%)
July	261	265	298	277	302	316	217	244	289	2469 (9.9%)
August	283	250	403	234	270	338	231	182	254	2445 (9.8%)
September	269	246	462	214	252	367	246	186	275	2517 (10.1%)
October	235	234	387	200	225	262	214	194	234	2185 (8.7%)
November	230	209	300	183	225	223	200	245	181	1996 (8.0%)
December	234	161	257	182	210	178	191	259	162	1834 (7.3%)
Gender										
Male	1869	1721	2209	1701	1799	1885	1543	1555	1742	16,024 (64.0%)
Female	1031	991	1180	935	973	1109	915	865	1000	8999 (36.0%)
Total number of patients	2900 (11.6%)	2712 (10.8%)	3389 (13.5%)	2636 (10.5%)	2772 (11.1%)	2994 (12.0%)	2458 (9.8%)	2420 (9.7%)	2742 (11.0%)	25,023

**Fig. 1:**
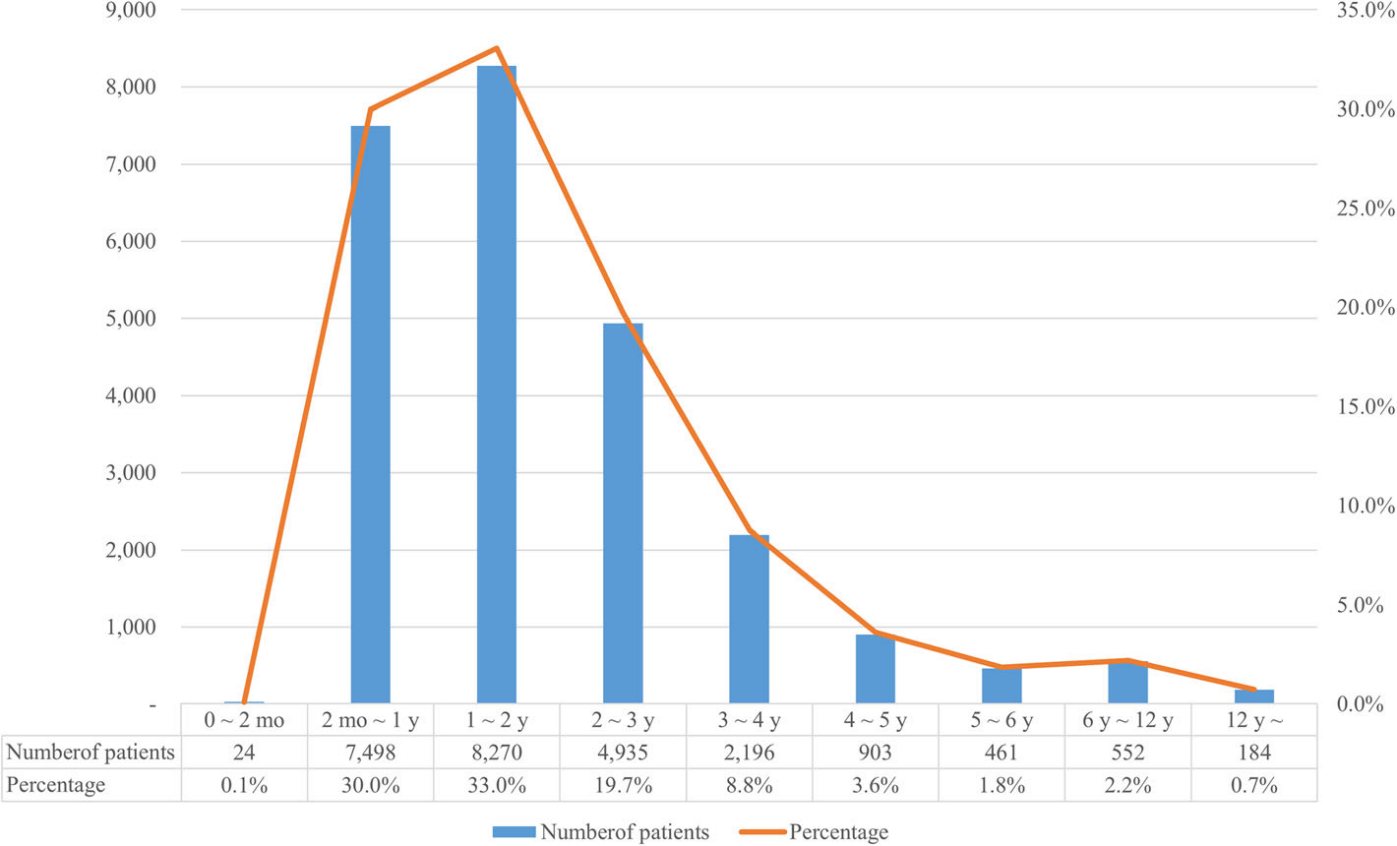
Distribution according to age among patients treated for intussusception in Korea from 2008 to 2016 (*P* = 0.053)

Figure 1 shows the number of intussusception patients by age (*P* = 0.053). There were 7498 patients (30.0%) between 2 and 12 months of age, 24 patients (0.1%) below 2 months, and 8270 patients (33.0%) between 1 and 2 years of age. The number of patients between 2 and 36 months of age was the highest at 20,703 (82.7%). Subsequently, the incidence gradually decreased with increasing age; 552 cases (2.2%) in the group aged 6 to 12 years, 184 cases (0.7%) in the adolescent age group of 12 to 18 years. The incidence of intussusception in children under 1 year of age was 193.2 per 100,000; South Korea’s cumulative population under 1 year of age from 2008 to 2016 was 3,892,954. The incidence per 100,000 people up to 2 years was 196.7 and 170.2 until the age of 3 years.

### Classification of comorbidities in patients treated for intussusception

**Fig. 2:**
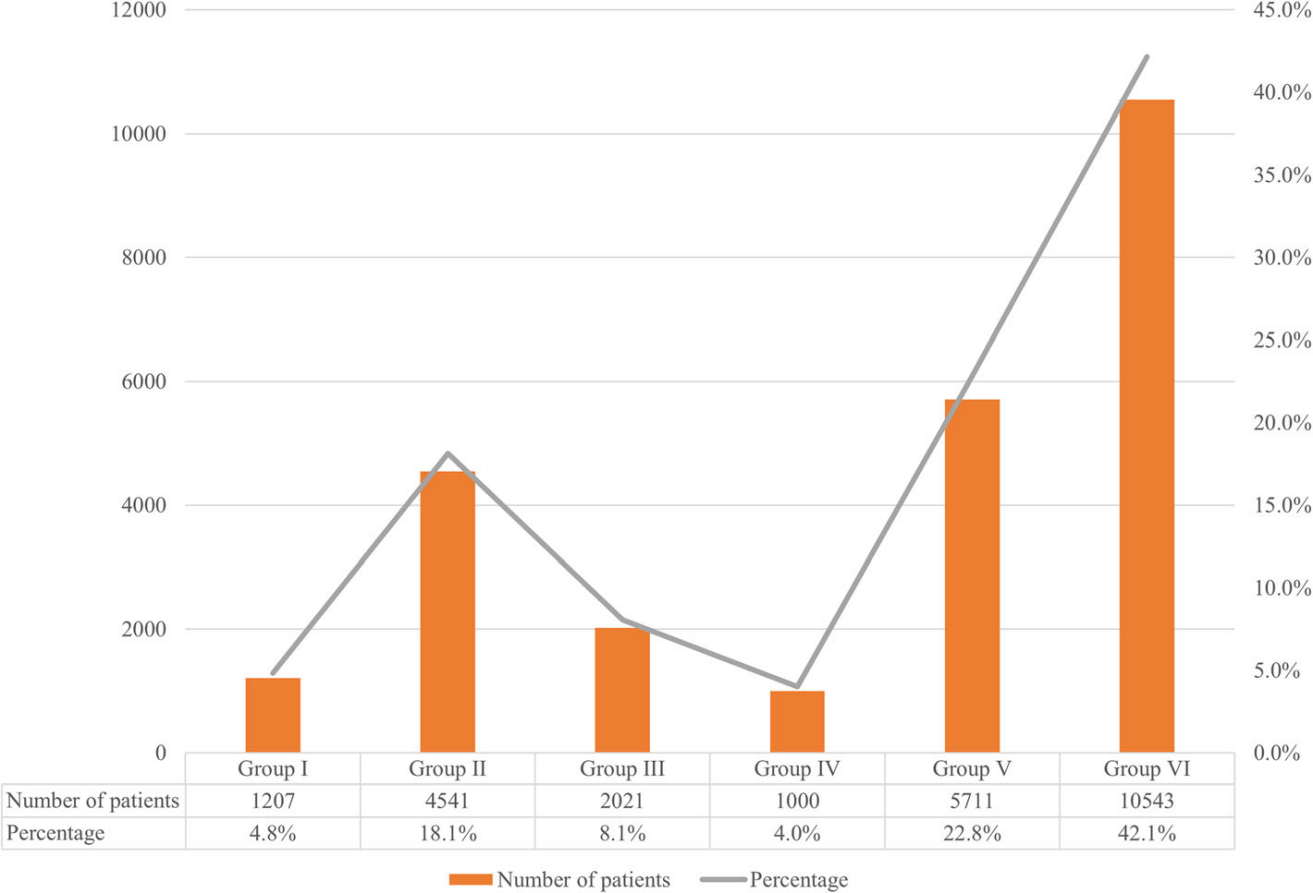
Classification of comorbidities in patients treated for intussusception in Korea from 2008 to 2016 (*P* = 0.200). Group I: Structural problem of the gastrointestinal tract, Group II: Acute gastrointestinal infectious disease, Group III: Acute respiratory infectious disease, Group IV: Other infectious disease, Group V: Systemic non-infectious disease, Group VI: No accompanying disease

The existing comorbidities in patients treated for intussusception are shown in Fig. 2 (*P* = 0.200). The number of patients with structural lesions in the gastrointestinal tract (group I), which could cause intussusception was 1207 (4.8%), while patients with acute gastrointestinal infectious disease (group II) such as acute enteritis and colitis were 4541 (18.1%). There were 2021 (8.1%) patients in group III with acute respiratory infectious disease such as influenza, and respiratory adenovirus. The patients with other infectious diseases (group IV) such as urinary tract infection and sepsis were 1000 (4.0%), and the number of group V patients with other non-infectious systemic diseases was 5711 (22.8%). Patients in whom no accompanying disease was registered were 10,543 (42.1%).

**Fig. 3:**
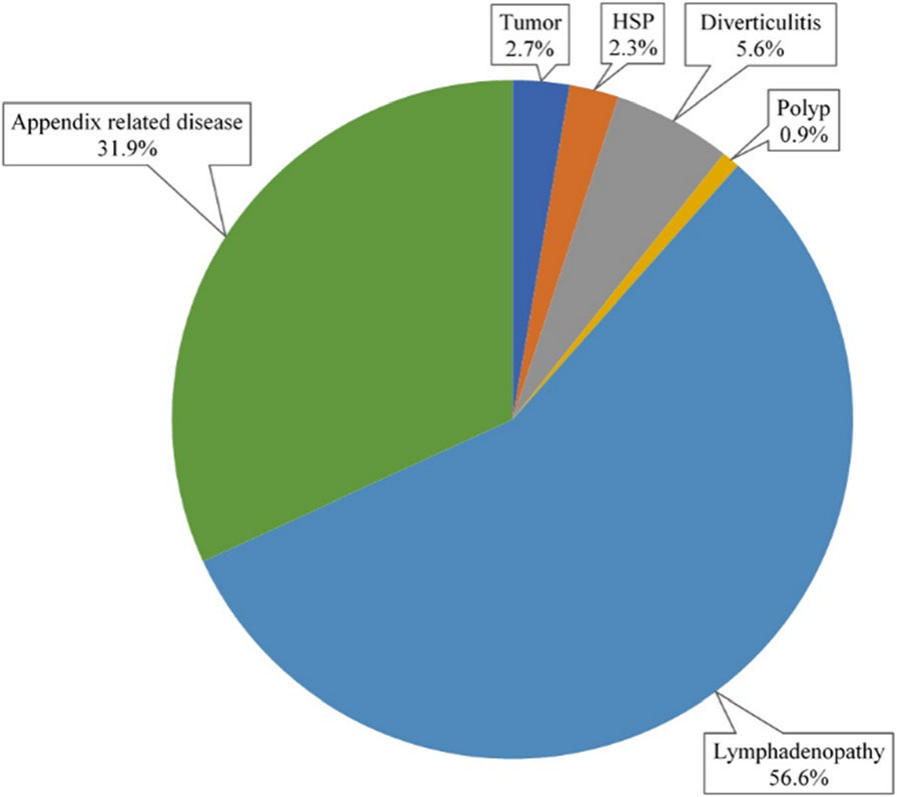
Classification of structural causes associated with intussusception in Korea from 2008 to 2016. HSP; Henoch–Schönlein purpura (*P* = 0.019). Of the 1207 patients with structural disease, 816 patients were classified into six disease groups and the remaining 391 patients had no specific problem

Among the structural lesions of the gastrointestinal tract, which could be the leading cause of intussusception, lymphadenopathy was the most common in 462 (56.6%) patients and an appendix related condition was seen in 260 (31.9%) patients while 391 patients had no specific lesion (Fig. 3) (*P* = 0.019). Number of patients with tumor like lymphoma were 22 (2.7%), and those with HSP, diverticulitis and polyp were 19 (2.3%), 46 (5.6%), and 7 (0.9%), respectively.

### Classification of comorbidity by age

**Fig. 4:**
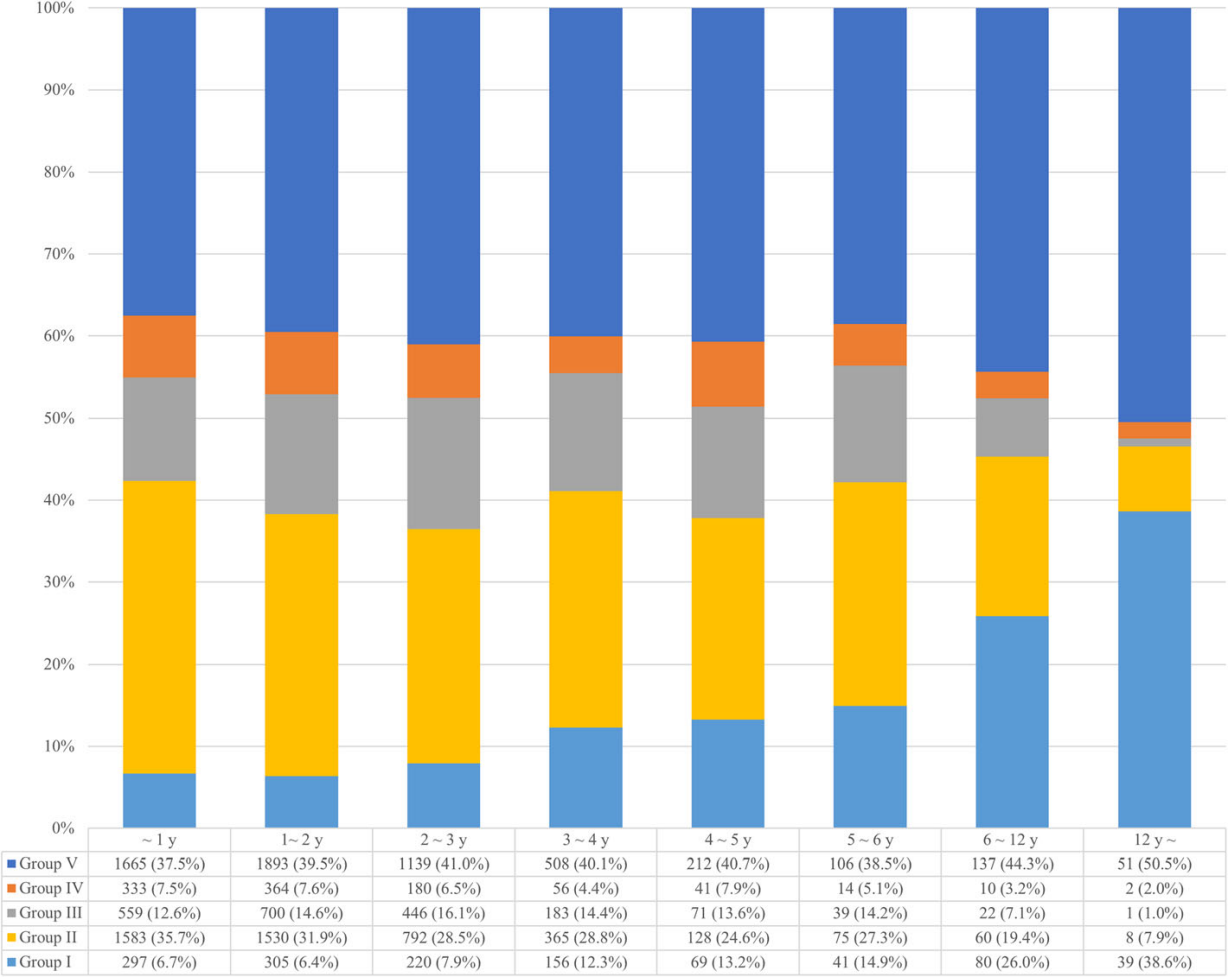
Classification of comorbidities according to age in patients treated for intussusception in Korea from 2008 to 2016 (*P* < 0.001). Group I: Structural lesion in the gastrointestinal tract, Group II: Acute gastrointestinal infectious disease, Group III: Acute respiratory infectious disease, Group IV: Other infectious disease, Group V: Systemic non-infectious disease, Group VI: No accompanying disease

The details of concomitant diseases according to age groups are shown in Fig. 4. Systemic non-infectious disease was the most frequently coexisting condition, cumulatively accounting for 40% across all age groups (*P* < 0.001). In patients under 6 years of age, acute gastrointestinal infectious disease was seen in about 30% of the cases, but the incidence sharply decreased in middle childhood above 6 years of age and in early adolescence above 12 years.

Structural problems of the gastrointestinal tract coexisting with intussusception were seen in less than 10% patients aged below 36 months but increased in those above 36 months and were almost 30% in middle childhood and 40% in early adolescence.

## Discussion

Since intussusception is fatal but not rare in pediatric patients, there are multiple studies and publications related to its cause and incidence [3, 7]. Although, previous researches there have been conducted on different races and regions, this is the first study that investigated the incidence throughout South Korea, using the national health database.

Previous reports have found the disease to be predominant in males; in the East Asian studies such as in China, Taiwan, and Japan, the ratio of male to female was reported to be 1.4 to 1.8: 1. However, previous studies from South Korea reported the male: female incidence to be 2.2 to 2.4: 1 [7, 16]. In our study, it was about 1.8:1 for 9 years and showed a similar ratio every year. The mortality rate varies greatly depending on the level of medical care, but the mortality rate of 0.02% was significantly lower compared to the previous reports. Hospitalized treatment was provided for 64.8% of the patients, and there was reduction failure in 3.18% of the patients. Since the treatment policy, medical accessibility, and the proficiency level of the medical staff vary by each medical institution, it is difficult to determine the degree of severity.

There are many reports of seasonal or monthly variation in the region, based on climatic variability. Previous studies, including those from East Asia are inconsistent; however, there are many reports about the intussusception rates being similar to the epidemic of acute gastroenteritis [17–19]. Our results showed that the rate of intussusception in South Korea was higher in the period from July to September, and there were fewer cases in the cold season. In addition, seasonal variation occurred 1.74 times higher in September, when the number of patients was the largest, compared to February, when the number of patients was the lowest. Although the pattern appeared to be similar year after year, the incidence was particularly high in August, September, and October in the year 2010. Due to the legal policies of the HIRA database, we could not access the patients’ personal information, but if we had been able to confirm if there was an epidemic of gastrointestinal tract or respiratory virus infection at the time, we could have provided a better insight on the probable etiology.

There are various reports about the peak age for intussusception. The highest peak is seen in the first year of life, but many studies have shown the peak to be from 5 months to 2 years of age [3]. One report mentioned that the peak incidence is during 3 to 36 months of age, because a relatively large number of cases occur between the age of 2 to 3 years [18, 20–24]. Our results also show that 63.1% of the patients were up to 24 months of age, and 19.7% of patients were 24 to 36 months of age; thus, 82.8% of patients were under 36 months of age. It is difficult to confirm accurately, but we think that the peak age for intussusception in South Korea is less than 36 months.

We investigated the diseases accompanying intussusception using the name and code of the disease registered in the HIRA database. However, due to limitations in the HIRA database, and the nature of intussusception, it is possible that some diseases associated with intussusception have not been captured. In case of acute infectious diseases, there might be cases in which the pathogen could not be confirmed, because either the patient was not investigated, or the treatment period was short. Concomitant diseases such as tumors can be identified in more cases if the diagnostic tests and diagnoses are almost certain, and a low rate of respiratory infection pathogens with simple or untested tests will be identified. Although, these infections do not directly induce intussusception, it is thought to be caused by the hypertrophy of lymph nodes and intestinal edema, which accompany the infection [25].

However, due to the nature of the disease, comorbidities that can become the leading cause are often investigated during a prolonged period of hospitalization, indicating most of the patients who have real diseases. In particular, malignancies such as lymphoma are thought to have a higher rate of reflection, but the relative rate is not as high as in other studies [26, 27]. Patients with a structural lesion were relatively few in those below 36 months of age, which is the most common age for intussusception, and the rate of structural lesions increased after 36 months of age. The highest rate of structural anomalies was found in the early adolescent period.

There are some limitations in our study. We identified 49,315 patients with intussusception as the chief or concomitant diagnosis, but patients who did not undergo air reduction or surgical methods were excluded. But, patients with intussusception will almost certainly be treated, so there will be no major problems. The rate of recurrence could not be determined because it was not clear whether the current episode was the first episode or a recurrence. Hence, the actual overall incidence might have been lower. Even when intussusception was confirmed, cases of self-reduction or small bowel intussusception that were only kept under observation have been excluded from the analysis. Moreover, as mentioned earlier, it is not known whether a comorbid condition directly caused intussusception. In the case of diseases such as lymphoma and polyp, they were included as a structural problem only if the disease was found at a location that could cause intussusception, such as the ileum or the colon. In such cases, the intussusception might not be a direct result of the structural lesion, but might merely co-occur. However, this number is expected to be negligible for a period of 9 years.

Many studies regarding the epidemiology of intussusception have been conducted in various countries [28–31]. Indeed, in many industrialized countries, the incidence of intussusception has decreased. If our study had been conducted over a period of 20 to 30 years, the results might have been more interesting. It is necessary to confirm whether the reduction of infectious diseases or the early diagnosis of other comorbidities is associated with a decrease in intussusception, since Korea has industrialized at a faster rate than any other country in the world and the level of medical care has improved significantly.

Despite the above limitations, this study was the first in South Korea to investigate the epidemiology of intussusception over a nine-year period using the national health database and to investigate the accompanying comorbidities that could be associated with the cause. We also analyzed the variations in concomitant diseases by age group. In conclusion, we confirmed that the occurrence of pediatric intussusception in South Korea shows a seasonal tendency, which is age-dependent and related to the exposure to infectious agents, and suggest that some infectious pathogens might play an important role in its pathophysiology. It is necessary to confirm the exact causal relationships through further long-term epidemiological studies.

## Data Availability

The data will not be shared because it will be used as material for other papers.

## Abbreviations

HIRA: Health Insurance Review and Assessment Service
HSP: Henoch-Schönlein purpura
ICD: International Classification of Disease
